# Factors Associated with Dental Care Utilization for Oral Disease Prevention Among Adolescents in Multicultural Families in Republic of Korea

**DOI:** 10.3390/healthcare12212141

**Published:** 2024-10-28

**Authors:** Seon-Hui Kwak, Deuk-Sang Ma

**Affiliations:** 1 Department of Dental Hygiene, College of Dentistry, Gangneung-Wonju National University, Gangneung 25457, Republic of Korea; ssun7681@gwnu.ac.kr; 2 Research Institute of Dental Hygiene Sciences, Gangneung-Wonju National University, Gangneung 25457, Republic of Korea; 3 Department of Preventive Dentistry, College of Dentistry, Gangneung-Wonju National University, Gangneung 25457, Republic of Korea; 4 Research Institute of Oral Sciences, Gangneung-Wonju National University, Gangneung 25457, Republic of Korea

**Keywords:** adolescents, dental care utilization, multicultural family, oral health behavior, preventive dental care

## Abstract

Objectives: This study utilized data from the 2022 Korea Youth Risk Behavior Survey to examine the utilization of preventive dental care among adolescents from multicultural families and analyze the associated factors. Methods: The number of adolescents from multicultural families, based on parental nationality, was 1361. The dependent variables were asymptomatic dental visits, sealant experience, and scaling experience. The independent variables included health perception factors (subjective health status, subjective oral health status), health behaviors (fruit consumption frequency, vegetable consumption frequency, sugary drink consumption frequency, drinking experience, smoking experience), and oral health behaviors (daily tooth-brushing frequency, tooth brushing after lunch, use of oral care products, oral symptom experience). Confounding variables included demographic variables (age, grade) and socioeconomic variables (academic performance, economic level, parental education level, mother’s nationality). Statistical analyses were conducted using a complex samples cross-tabulation and complex samples logistic regression. Results: Asymptomatic dental visits were significantly associated with subjective oral health status and sugary drink consumption. Those who perceived their oral health as “Healthy” had an odds ratio (OR) of 3.34 (CI = 1.76–6.32; *p* < 0.001), and those who perceived themselves “Normal” had an OR of 2.03 (CI = 1.08–3.82; *p* = 0.029). A sugary drink consumption of less than three times per week was linked with an OR of 1.68 (CI = 1.05–2.70; *p* = 0.031). Sealant experience was associated with brushing before bedtime (OR = 2.27, CI = 1.18–4.35; *p* = 0.014) and using more than one oral care product (OR = 1.97, CI = 1.27–3.07; *p* = 0.003). Scaling experience associated with oral symptoms (OR = 1.94, CI = 1.21–3.11; *p* = 0.006). Conclusions: To enhance access to preventive dental care utilization for adolescents from multicultural families, efforts are needed to raise subjective oral health awareness, improve oral health behaviors, and develop strategies that ensure timely preventive dental care.

## 1. Introduction

A multicultural family in Republic of Korea was defined as a household consisting of a Korean national and individuals who had acquired Korean citizenship through marriage, birth, or naturalization [1]. This definition also included families formed by individuals who obtained Korean citizenship through acknowledgment or naturalization. Multicultural families were characterized by cultural diversity, encompassing various cultural backgrounds, values, traditions, religions, and lifestyles.

As Republic of Korea underwent rapid economic development and social changes due to globalization, issues such as marriage imbalance in low-income urban areas and rural regions, low birth rates, and population aging emerged. To address these issues, an influx of foreign workers and international marriages in the 1990s led to a steady increase in the number of married female immigrants from countries such as Vietnam, Philippines, and Thailand, which resulted in a growing number of multicultural families [2]. In recent years, the number of adolescents from multicultural families increased from 46,000 in 2012 to 181,000 in 2022 due to a rise in intermarriages, and the number is expected to increase further [3].

According to a report by the Ministry of Health and Welfare [4], 52.9% of multicultural families with married female immigrants live below the minimum cost of living, underscoring their low socioeconomic status. Many individuals in these families face not only economic and language barriers but also geographic challenges due to residing in rural or small urban areas. Consequently, they often encounter difficulties in accessing social welfare services and dental care, frequently remaining unaware of the existence of such services [4]. Additionally, the long-standing history of a homogeneous society renders these groups vulnerable to social discrimination. In response, the government has classified multicultural families as a socially disadvantaged group and actively sought strategies to ensure that members of these families received equal welfare and benefits compared to their non-multicultural families [5].

The barriers to dental care utilization faced by multicultural families also impact their children’s access to dental services [6]. Adolescents from multicultural families in Republic of Korea face a higher likelihood of limited access to preventive oral health care, not only due to the financial burden on their parents but also because of the linguistic and cultural communication barriers encountered by foreign parents, which has been reported to be high [4,6]. An investigation into the preventive dental care of adolescents from multicultural families revealed that only 24.0% had experienced scaling, and 25.5% had received sealants. In comparison, 32.6% of non-multicultural adolescents had undergone scaling, and 26.9% had received sealants, indicating a lack of access to preventive dental care among adolescents from multicultural families [7].

Adolescence represents a critical period during which teeth erupt and mature, resulting in a heightened risk of dental caries. This phase also marks the onset of periodontal disease due to hormonal changes and oral risk behaviors [8]. Establishing proper oral health behaviors during this time can prevented oral diseases and significantly influence lifelong oral health outcomes [9].

At this crucial juncture, there is a need for sustained efforts to reduce disparities and achieve health equality among Korean adolescents. Specifically, it is essential to improve access to preventive oral care for adolescents from multicultural families who belong to socioeconomically vulnerable groups. Furthermore, multifaceted initiatives are necessary to identify the factors associated with oral disease prevention and oral health promotion among these adolescents, as well as to develop appropriate strategies to ensure the provision of essential preventive dental services without gaps [10].

According to previous studies, adolescent health behaviors were reported to be associated with a variety of factors, including household income, parental education, family support, peer support, and health literacy [11,12,13,14]. Additionally, it was reported that socioeconomic status, along with differences in appearance, language, and parents’ country of origin and culture, was associated with the oral health behaviors and status of adolescents from multicultural families, such as subjective oral health status, oral symptoms, smoking, alcohol use, and drug use [15,16,17].

However, previous studies primarily focused on identifying factors related to the subjective oral health status or self-care behaviors of adolescents from multicultural families [18]. Given the contemporary emphasis on the necessity of preventive dental care—such as dental examinations, sealants, and scaling—for the prevention of oral diseases during adolescence, which has been recommended globally [19,20], there remains a lack of research investigating the factors associated with preventive dental care utilization among multicultural families in Republic of Korea.

Therefore, the aim of the present study was to employ data from the Korea Youth Risk Behavior Survey (KYRBS) to evaluate the utilization of preventive dental care among adolescents from multicultural families in Republic of Korea and identify the factors associated with this utilization.

The hypothesis of this study was that the utilization of preventive dental care among adolescents from multicultural families was associated with health perception factors, health behavior factors, and oral health behavior factors, after adjusting for demographic and socioeconomic factors.

## 2. Methods

### 2.1. Study Design and Ethical Considerations

This study was a cross-sectional analysis of secondary data. This study was approved by the Institutional Review Board of the Gangneung-Wonju National University Dental Hospital (GWNUDH-IRB2023-A006) to conduct secondary data analyses based on the results of the survey, with informed consent from participants.

### 2.2. Data Sources

This study analyzed data from the 2022 KYRBS to identify the factors associated with preventive dental care utilization among adolescents from multicultural families in Republic of Korea. The KYRBS is a nationally recognized statistical survey conducted annually among students from the first grade of middle school to the third grade of high school to understand the health behaviors of Republic of Korean adolescents in 15 areas, including physical activity, diet, and oral health [7]. Additionally, the KYRBS obtains consent regarding the provision of household information and collects data on the nationalities of both fathers and mothers of students who consent to participate in the survey; therefore, it was considered an appropriate survey for this study. After approval from the Korea Disease Control and Prevention Agency (KDCA), we downloaded and used the raw data of the 2022 KYRBS from the KDCA website.

### 2.3. Study Population

Participants in the KYRBS from middle and high schools were classified to be from multicultural families if they consented to the household survey and had at least one non-Korean parent. This analysis included a total of 1361 students.

### 2.4. Variable Selection

#### 2.4.1. Dependent Variables

To identify factors influencing preventive dental care utilization, the dependent variables were asymptomatic dental visit experience, sealant experience, and scaling experience.

Asymptomatic dental visit experience was established based on the responses to two questions: “Have you experienced any oral symptoms in the last 12 months?” and “Have you had any dental care (including oral examinations at a dental or health clinic) in the last 12 months?”. Respondents who answered “No” to all listed symptoms—such as broken or chipped teeth, tooth pain while consuming hot or cold beverages or food, persistent toothache, gum pain or bleeding, tingling or soreness of the tongue or the inner cheek, or halitosis—and simultaneously answered “Yes” to having received dental care at least once in the last 12 months were included in this category.

The experience of sealants was analyzed based on the response to the question “Have you received sealants in the last 12 months?”. Participants who responded “Yes” were considered to have experience and were included in the analysis. Similarly, the experience of scaling was analyzed using the response to the question “Have you received scaling in the last 12 months?”. Only those who answered “Yes” were regarded as having experience and were included in the analysis.

#### 2.4.2. Confounding Variables

Demographic and socioeconomic factors have been reported to be associated with various health behaviors in adolescents, including smoking, drinking, physical activity, tooth brushing, and dental care utilization [21,22,23]. Considering this, demographic and socioeconomic factors were set as confounding variables, and a complex samples logistic regression analysis was conducted to adjust for these factors.

Demographic factors included sex and grade. Sex was categorized as “Male” and “Female”. The grade variable was utilized as a variable that could substitute for age; in the Republic of Korean educational system, a 1st-year middle school student is typically 13 years old, while a 3rd-year high school student is generally considered to be 18 years old. The findings were derived from the response to the question “What grade are you in?”. Grades were categorized as follows: “1st year of middle school”, “2nd year of middle school”, “3rd year of middle school”, “1st year of high school”, “2nd year of high school”, and “3rd year of high school”.

Socioeconomic factors included academic performance, subjective economic status, father’s and mother’s education, and mother’s nationality. Academic performance was assessed using the question “How has your academic performance been over the past 12 months?”. Responses indicating “high” or “moderate–high” were classified as “High”, responses indicating “middle” were classified as “Middle”, and responses indicating “moderate–low” or “low” were classified as “Low”.

Subjective economic status was determined using the question “How would you describe your family’s economic status?”. Responses indicating “high” or “moderate–high” were classified as “High”, responses indicating “middle” were classified as “Middle”, and responses indicating “moderate–low” or “low” were classified as “Low”.

The father’s and mother’s education levels were determined through the questions “What is your father’s highest level of education?” and “What is your mother’s highest level of education?”. Responses indicating “middle school graduate or below” or “high school graduate” were classified as “Less than a high school graduate”, while responses indicating “college graduate or higher (including those with vocational college degrees)” were classified as “College degree or higher”. Responses of “I don’t know” were treated as missing values. The variance inflation factor of the father’s and mother’s education was 1.000. Accordingly, both variables were considered suitable for the model and were included in the complex logistic regression analysis.

A mother’s nationality was determined using the question “In which country was your mother born?”. Considering that most women who immigrate to Republic of Korea as marriage migrants are from China, Vietnam, and the Philippines, the focus was primarily on the proportions of these nationalities. Responses indicating “North Korea”, “Japan”, “Taiwan”, “Mongolia”, “Thailand”, “Cambodia”, “Uzbekistan”, “Russia”, and “other nationalities” had low response rates and were categorized as “Other”.

#### 2.4.3. Independent Variables

The independent variables were categorized into health perception, health behavior, and oral health behavior factors to analyze the multifaceted factors.

Health perception factors included subjective health status and subjective oral health status, as subjective health status significantly affects adolescent health behaviors [24]. Subjective health status was evaluated using the question “How would you rate your overall health?”. Responses indicating “very healthy” or “healthy” were classified as “Healthy”, responses indicating “moderate” were classified as “Normal”, and responses indicating “unhealthy” or “very unhealthy” were classified as “Unhealthy”.

Subjective oral health status was evaluated using the question “How would you rate your oral health, including your teeth and gums?”. Responses indicating “very good” or “good” were classified as “Healthy”, responses indicating “moderate” were classified as “Normal”, and responses indicating “poor” or “very poor” were classified as “Unhealthy”.

Health behavior factors encompassed variables related to diet, alcohol consumption, and smoking, which are known factors affecting oral health. Dietary behaviors included the frequency of fruit and vegetable consumption, which positively affects oral health, and sugary drink consumption, which negatively affects oral health.

Fruit consumption was determined using the question “In the past 7 days, how often did you consume fruit (excluding fruit juice)?”. Responses indicating “did not consume in the past 7 days”, “1–2 times per week”, “3–4 times per week”, or “5–6 times per week” were classified as “Less than once per day”. In contrast, responses indicating “once daily” or “twice daily” or “three times or more daily” were classified as “At least once per day”. Vegetable consumption was evaluated using the question “In the past 7 days, how often did you consume vegetables (excluding kimchi)?”. The response categories for vegetable consumption were applied in the same manner as those for fruit consumption. Sugary drink consumption was assessed using the question “In the past 7 days, how often did you consume sweetened beverages?”. Responses indicating “did not consume in the past 7 days” or “1–2 times per week” were classified as “Less than 3 times per week”. Conversely, responses indicating “3–4 times per week”, “5–6 times per week”, “once daily”, “twice daily”, or “three times or more daily” were classified as “At least 3 times per week”.

For drinking and smoking, the variables were categorized as alcohol drinking experience and smoking experience. Alcohol drinking experience was assessed using the question “Have you ever consumed more than one alcoholic beverage?”. Smoking experience was evaluated with the question “Have you ever smoked even a few puffs of a regular cigarette?”. Responses for both questions were recorded as “Yes” or “No”.

Oral health behavioral factors included tooth-brushing habits, use of oral care products, and oral symptoms. Tooth-brushing behavior included daily brushing frequency, brushing before bedtime, and brushing after lunch. Daily brushing frequency was assessed using the question “How many times did you brush your teeth yesterday?”. Responses indicating “0 times” or “1 time” were classified as “Less than 1 time”, while responses ranging from “2 times” to “9 times or more” were classified as “More than 2 times”. Brushing before bedtime was determined using the question “Did you brush your teeth before going to bed yesterday?”. Responses were categorized as “Yes” or “No”. Responses indicating “I didn’t sleep” were treated as missing values.

The use of oral care products was defined as the use of at least one of the following: dental floss, interdental toothbrush, or mouthwash. Experience of oral symptoms was assessed using the question “In the past 12 months, have you experienced any of the following symptoms?”. A response of “Yes” was classified if the participant reported experiencing at least one of the following symptoms: broken or chipped teeth, pain when consuming cold or hot food or beverages, toothache or sensitivity, gum pain or bleeding, discomfort or pain in the tongue or inside of the cheek, or unpleasant breath. The information regarding the variables was provided in the Supplementary Materials.

### 2.5. Data Analysis

Statistical analyses were performed using SPSS (version 28.0; SPSS Inc., Chicago, IL, USA). A complex samples cross-analysis was conducted to examine the relationship between preventive dental care experience in adolescents from multicultural families and socioeconomic and related factors. Furthermore, to explore the associations with health perception, health and oral health behaviors, and the impact of related factors in more depth, significant variables were input, and a complex samples logistic regression analysis was performed. The results of the complex samples cross-tabulation analysis expressed weighted numbers and percentage (%), while the results of the complex samples logistic regression analysis expressed odds ratios and 95% confidence intervals. Statistical significance was set at *p* < 0.05.

## 3. Results

### 3.1. General Characteristics of the Study Participants

The general characteristics of the Korean adolescents from multicultural families are presented in Table 1. Among the adolescents who participated in the 2022 KYRBS, 1361 (3.0%) were from multicultural families. These adolescents exhibited the highest rate of low academic performance at 40.6%. In terms of economic status, 26.6% were classified as high, 52.1% as middle, and 21.3% as low. Regarding maternal nationality, 41.1% of the mothers were Chinese, 27.5% were Vietnamese, and 8.6% were Filipino.

### 3.2. Association Between General Characteristics and Preventive Dental Care Utilization Among Adolescents from Multicultural Families

The association of preventive dental care utilization with the general characteristics of Korean adolescents from multicultural families is shown in Table 2. Among students who perceived their subjective oral health status as “Healthy”, 213 (26.6%) had experienced an asymptomatic dental visit, compared to 60 (15.8%) adolescents with a “Normal” status and 18 (11.5%) with an “Unhealthy” status (*p* < 0.001). In terms of health behavior, 57 adolescents (28.2%) who consumed at least one serving of fruit per day had experienced an asymptomatic dental visit, compared to 234 adolescents (20.4%) who consumed less than one serving per day (*p* = 0.028). Additionally, among adolescents who consumed sugary drinks fewer than two times per week, 130 (26.1%) had experienced asymptomatic dental visits, compared to 161 (18.9%) who consumed sugary drinks three or more times per day (*p* = 0.005).

Sealant experience was associated with health behavior factors and oral health behavior factors. Among adolescents who consumed vegetables at least once a day, 123 (30.9%) had experienced sealants, while among those who consumed vegetables less than once a day, 208 (22.8%) had experienced sealants (*p* = 0.002). In terms of oral health behaviors, brushing before bedtime (*p* < 0.001) and brushing after lunch (*p* = 0.005) were associated with a higher sealant experience. In addition, the use of at least one oral care product (*p* < 0.001) and experiencing oral symptoms (*p* = 0.024) were associated with a higher sealant experience.

Scaling experience was associated with health perception factors, health behavior factors, and oral health behavior factors. Among students with a subjective oral health status perceived as “Healthy”, 185 (23.0%) had experienced scaling, while among those perceived as “Unhealthy”, 43 (28.9%) had experienced scaling (*p* < 0.001). In terms of health behavior factors, 105 (28.2%) students with a history of alcohol consumption had experienced scaling. In terms of oral health behaviors, scaling experience was associated with brushing before bedtime (*p* = 0.011) and after lunch (*p* = 0.032). Scaling was associated with the use of more than one oral care product (*p* = 0.001) and experience of oral symptoms (*p* < 0.001).

### 3.3. Factors Associated with Preventive Dental Care Utilization Among Adolescents from Multicultural Families

Table 3 shows the results of the factors associated with preventive oral care utilization among adolescents from multicultural families, adjusted for demographic and socioeconomic factors.

Asymptomatic dental visits were found to have a significant association with subjective oral health status and sugary drink consumption. For subjective oral health status, the adjusted odds ratio (OR) of “Healthy” was 3.34 (confidence interval [CI] = 1.76–6.32; *p* < 0.001), while the OR of “Normal” was 2.03 (CI = 1.08–3.82; *p* = 0.029). Regarding sugary drink consumption, the OR of “Less than 3 times per week” was 1.68 (CI = 1.05–2.70; *p* = 0.031).

Sealant experience was found to have a significant association with brushing before bedtime and the use of oral care products. For brushing before bedtime, the OR of “Yes” was 2.27 (CI = 1.18–4.35; *p* = 0.014). Regarding the use of oral care products, the OR of “Use more than one” was 1.97 (CI = 1.27–3.07; *p* = 0.003).

Scaling experience was found to have a significant association with the experience of oral symptoms. For those who reported “Yes” to experiencing oral symptoms, the OR was 1.94 (CI = 1.21–3.11; *p* = 0.006).

## 4. Discussion

Republic of Korea experienced rapid economic growth and various social changes, leading to an increase in the influx of foreign workers and marriage migrants, which consequently resulted in a rise in the number of multicultural families. However, these individuals have been reported to face limitations in accessing dental care due to geographic, economic, and linguistic barriers [25]. Recent studies have demonstrated that the dental care utilization of parents from multicultural families was closely associated with their children’s health management [6,26]. Notably, adolescence is a critical period characterized by vulnerability to dental caries and periodontal diseases, which necessitates comprehensive preventive dental care. Nevertheless, it was reported that adolescents from multicultural families in Republic of Korea had limited access to preventive care services [27].

To address this issue and enhance the oral health of adolescents from multicultural families, it is crucial to develop and implement health promotion programs and policies that consider their specific characteristics. This approach needs to be grounded in research identifying the oral-health-related issues these adolescents face. Given the significant role of dental care in maintaining oral health, investigating the factors associated with the utilization of preventive dental care could potentially increase its uptake, promote oral health maintenance, and reduce disparities among adolescents from multicultural families [11,28]. Therefore, the purpose of this study was to assess the current status of preventive oral care among adolescents from multicultural families and to identify the related factors, thereby providing the foundational data essential for developing oral health policies tailored to this demographic. This study supported the hypothesis that oral health perception factors, health behavior factors, and oral health behavior factors were associated with preventive dental care among adolescents from multicultural families.

When adjusted for demographic and socioeconomic factors, adolescents from multicultural families who perceived their oral health as healthy were three times more likely to have visited a dentist, even without experiencing symptoms. These findings aligned with Park’s research [29], which suggested that individuals who positively perceived their oral health and understood the benefits of maintaining good oral hygiene were more inclined to engage in preventive oral health behaviors. Regular visits to dentists for preventive or routine professional care, even in the absence of symptoms, were crucial for preventing oral diseases and promoting oral health. These visits enabled professionals to monitor oral health risks and educate individuals on preventive care [30]. In fact, the National Health Service (NHS England) recommended that adolescents aged 7–19 years undergo professional dental checkups every 3–12 months, depending on their oral health status [20]. Given the significance of subjective oral health perception associated with these behaviors, efforts should be directed toward increasing awareness among adolescents from multicultural families regarding the importance of oral healthcare. This includes educating patients about the prevention of oral diseases, emphasizing the necessity of regular dental checkups, and providing guidance on accessing preventive dental care. Such initiatives could potentially increase dental care utilization among adolescents from multicultural families in the future. Given the need for a greater perception for preventive oral care among parents, who are closely associated with the actual implementation of preventive care for their children [31,32], it is essential to provide continuous education and establish an environment that encourages mothers from multicultural families to recognize the importance of preventive care, starting during pregnancy and continuing throughout their children’s upbringing. Such efforts might enhance the likelihood of preventive oral care practices being passed on to adolescents, thereby increasing their utilization of dental services [33].

Sealant experience was associated with oral hygiene behaviors, such as brushing before bedtime and the use of oral care products. In contrast, scaling experience was associated with the experience of oral symptoms. These results were consistent with a previous study by Park et al. [34], who observed that daily tooth brushing correlated with increased sealant use to prevent oral diseases. Jeon and Kim [35] reported that adolescents sought scaling treatment when they experienced oral issues such as gum pain and bleeding. Park et al. [34] argued that adolescents practicing good oral hygiene through self-care were less susceptible to dental caries and more likely to be recommended sealant applications by oral health professionals; therefore, efforts should be made to improve adolescents’ self-care skills. Park et al. [34] and Kwak [36] noted that adolescents sought scaling in response to oral symptoms, highlighting the need for strategies encouraging preventive scaling before symptoms manifest. Considering the potential socioeconomic burden that could prevent access to dental care, it is essential to ensure financial support for preventive treatments, such as sealants and scaling, for economically disadvantaged adolescents from multicultural families through the National Health Insurance system. In Republic of Korea, sealants are covered by health insurance for individuals under 18 years old, resulting in a personal cost of approximately USD 7 (10% copayment). However, scaling is covered by health insurance only for adults aged 19 and older, leading to a personal cost of approximately USD 38 to USD 53 for adolescents not covered by insurance. Therefore, it is necessary to lower the copayment for sealants and extend health insurance coverage for scaling to improve financial access.

Regular oral examinations, sealants, and scaling are recognized as highly cost-effective interventions for preventing dental caries and periodontal diseases. Based on the findings of this study, enhancing the utilization of dental care to prevent oral diseases among adolescents from multicultural families requires strategic initiatives. First, it is essential to provide regular oral health education aimed at improving awareness and self-care skills among adolescents from multicultural families. Additionally, establishing and implementing comprehensive preventive care programs is crucial for ensuring timely access to services such as sealants and scaling. However, in developing preventive care programs tailored for adolescents from multicultural families, it is critical to acknowledge the barriers they face, including limited access to healthcare due to their parents’ language and cultural differences [6]. Overcoming these barriers necessitates the active involvement of both parents and adolescents in preventive care programs. This involvement might include the use of customized oral health education materials tailored to the nationality and language preferences of parents. Addressing the potential gaps in parents’ knowledge, behaviors, and perceptions regarding oral health, which might arise from their cultural backgrounds, appears to be significant [37,38]. Cultural differences in health beliefs likely influence how parents perceive the importance of preventive dental care for their children. For instance, some cultural backgrounds might prioritize alternative medicine or hold differing interpretations of oral health, which could lead to varying levels of engagement with conventional dental practices. Recognizing these cultural beliefs is crucial for developing effective health promotion strategies. Additionally, language barriers likely pose challenges for many parents in accessing essential information about oral health due to limited proficiency. This situation could result in misunderstandings about recommended preventive dental care. By providing educational materials in multiple languages and formats that consider the cultural contexts of these families, health programs might bridge the gap in understanding and encourage proactive health behaviors among parents and their children. Therefore, to enhance the understanding and involvement of parents from diverse foreign backgrounds in implementing preventive oral health care for their children, dental care professionals need to collaborate with experts from various fields and establish comprehensive intervention and support strategies.

While this study has limitations, such as not fully considering cultural and environmental factors and relying on cross-sectional secondary data, it provides foundational insights for developing effective strategies to promote oral health and address disparities in the multicultural population. This study is novel in that it specifically addresses the utilization of preventive dental care among adolescents from multicultural families in Republic of Korea, a topic that has not been thoroughly explored in prior research. Whereas previous studies have primarily investigated disparities in oral health behaviors, such as tooth brushing, this study provides a more comprehensive understanding of the barriers multicultural adolescents face by considering health perception factors, health behavior factors, and oral health behavior factors concurrently. Furthermore, by analyzing nationally representative data, this study offers substantial evidence that could inform the development of tailored policy interventions aimed at reducing oral health disparities in this vulnerable population.

## 5. Conclusions

This study revealed significant associations between health perception factors, health behaviors, and oral health behaviors in the preventive dental care of adolescents from multicultural families. Consequently, to enhance access to oral health care services for the prevention of oral diseases and the promotion of oral health among adolescents from multicultural families, it is critical to raise awareness regarding the necessity of oral disease prevention, improve health behaviors such as dietary habits and tooth-brushing practices, and develop comprehensive oral health promotion strategies tailored to this demographic, ensuring timely and effective preventive care.

## Figures and Tables

**Table 1:** General characteristics of the study participants (Unit: N (wt%)).

Variables		Multicultural	Non-Multicultural
Total		1361 (3.0)	36,959 (97.0)
Demographics	Sex		
	Male	644 (48.1)	17,594 (48.2)
	Female	717 (51.9)	19,365 (51.8)
	Grade		
	1st year of middle school	410 (26.5)	7312 (18.5)
	2nd year of middle school	291 (19.6)	6955 (17.9)
	3rd year of middle school	272 (19.6)	6768 (18.2)
	1st year of high school	146 (12.5)	5916 (16.0)
	2nd year of high school	125 (9.5)	5278 (14.3)
	3rd year of high school	117 (12.2)	4730 (14.9)
Socioeconomic factors	Academic performance		
	High	388 (28.0)	15,139 (41.0)
	Middle	438 (31.3)	11,077 (30.1)
	Low	535 (40.6)	10,743 (28.8)
	Economic status		
	High	353 (26.6)	16,534 (45.6)
	Middle	724 (52.1)	17,013 (45.5)
	Low	284 (21.3)	3412 (8.9)
	Father’s education		
	Less than a high school graduate	554 (63.1)	7663 (23.6)
	College degree or higher	303 (36.9)	22,912 (76.4)
	Mother’s education		
	Less than a high school graduate	448 (57.0)	8575 (26.2)
	College degree or higher	325 (43.0)	22,679 (73.8)
	Mother’s nationality		
	China	478 (41.1)	-
	Vietnam	409 (27.5)	-
	Philippines	126 (8.6)	-
	Other	268 (22.8)	-

**Table 2:** Prevalence of preventive dental care utilization according to health and oral health factors of adolescents from multicultural families (Unit: N (wt%)).

Variables		Total	Experience of an Asymptomatic Dental Visit	*p* *	Sealant Experience	*p* *	Scaling Experience	*p* *
Health perception factors	Subjective health status							
	Healthy	819 (59.2)	213 (26.6)	<0.001	203 (26.0)	0.486	185 (23.0)	0.341
	Normal	383 (27.9)	60 (15.8)		87 (23.2)		85 (23.8)	
	Unhealthy	159 (12.9)	18 (11.5)		41 (28.0)		43 (28.9)	
	Subjective oral health status							
	Healthy	288 (22.3)	92 (31.9)	<0.001	56 (21.5)	0.199	48 (15.4)	<0.001
	Normal	679 (47.4)	162 (24.1)		168 (25.9)		162 (25.3)	
	Unhealthy	394 (30.4)	37 (10.1)		107 (27.8)		103 (28.2)	
Health behavior factors	Fruit consumption							
	At least once per day	210 (15.4)	57 (28.2)	0.028	63 (29.3)	0.189	49 (21.5)	0.394
	Less than once per day	1149 (84.6)	234 (20.4)		267 (24.8)		263 (24.3)	
	Vegetable consumption							
	At least once per day	449 (32.7)	105 (23.7)	0.212	123 (30.9)	0.002	106 (25.7)	0.336
	Less than once per day	912 (67.3)	186 (20.6)		208 (22.8)		207 (23.1)	
	Sugary drink consumption							
	Less than 2 times per week	525 (38.0)	130 (26.1)	0.005	120 (24.3)	0.424	112 (21.6)	0.145
	At least 3 times per week	836 (62.0)	161 (18.9)		211 (26.3)		201 (25.4)	
	Alcohol drinking experience							
	No	955 (69.5)	219 (23.5)	0.018	232 (24.4)	0.203	208 (22.1)	0.021
	Yes	406 (30.5)	72 (17.4)		99 (28.0)		105 (28.2)	
	Smoking experience							
	No	1240 (90.7)	265 (21.5)	0.717	309 (26.0)	0.228	290 (24.5)	0.138
	Yes	121 (9.3)	26 (23.0)		22 (20.3)		23 (18.4)	
Oral health behavior factors	Daily brushing frequency							
	Less than 1 time	225 (16.7)	26 (12.4)	<0.001	46 (22.7)	0.338	39 (19.6)	0.154
	More than 2 times	1136 (83.3)	265 (23.5)		285 (26.0)		274 (24.8)	
	Brushing before bedtime							
	Yes	1135 (84.0)	255 (22.5)	0.138	293 (27.2)	<0.001	276 (25.4)	0.011
	No	211 (16.0)	34 (17.3)		31 (14.4)		35 (16.5)	
	Brushing after lunch							
	Yes	461 (31.3)	118 (25.0)	0.043	135 (31.2)	0.005	130 (28.1)	0.032
	No	900 (68.7)	173 (20.1)		196 (22.9)		183 (22.1)	
	Use of oral care products							
	Use more than one	664 (50.0)	154 (23.4)	0.164	138 (30.9)	<0.001	186 (28.4)	0.001
	Disabled	697 (50.0)	137 (19.8)		138 (20.1)		127 (19.5)	
	Experience of oral symptoms							
	Yes	848 (62.7)	0 (0.0)	<0.001	230 (27.7)	0.024	230 (28.9)	<0.001
	No	513 (37.3)	291 (58.0)		101 (21.8)		83 (15.7)	

* The data were analyzed by a complex samples Chi-square test.

**Table 3:** Factors influencing preventive dental care utilization among adolescents from multicultural families (Unit: Odds ratio (95% confidence interval)).

Variables		Experience of an Asymptomatic Dental Visit	*p* *	Sealant Experience	*p* *	Scaling Experience	*p* *
Health perception factors	Subjective health status						
	Healthy	1.64 (0.75–3.60)	0.213	-	-	-	-
	Normal	0.92 (0.41–2.09)	0.847	-		-	
	Unhealthy	1.00		-		-	
	Subjective oral health status						
	Healthy	3.34 (1.76–6.32)	<0.001	-	-	0.61 (0.34–1.09)	0.096
	Normal	2.03 (1.08–3.82)	0.029	-		0.82 (0.52–1.29)	0.386
	Unhealthy	1.00		-		1.00	
Health behavior factors	Fruit consumption						
	At least once per day	1.25 (0.71–2.21)	0.434	-	-	-	-
	Less than once per day	1.00		-		-	
	Vegetable consumption						
	At least once per day	-	-	1.37 (0.91–2.06)	0.126	-	-
	Less than once per day	-	-	1.00		-	
	Sugary drink consumption						
	Less than 2 times per week	1.68 (1.05–2.70)	0.031	-	-	-	-
	At least 3 times per week	1.00		-		-	
	Alcohol drinking experience						
	No	1.16 (0.72–1.87)	0.533	-	-	0.86 (0.58–1.26)	0.431
	Yes	1.00		-		1.00	
	Smoking experience						
	No	-	-	-	-	-	-
	Yes	-		-		-	
Oral health behavior factors	Daily brushing frequency						
	Less than 1 time	1.50 (0.71–3.16)	0.290	-	-	-	-
	More than 2 times	1.00		-		-	
	Brushing before bedtime						
	Yes	-	-	2.27 (1.18–4.35)	0.014	1.46 (0.82–2.61)	0.195
	No	-		1.00		1.00	
	Brushing after lunch						
	Yes	1.51 (0.94–2.40)	0.086	1.07 (0.68–1.71)	0.766	1.02 (0.65–1.60)	0.926
	No	1.00		1.00		1.00	
	Use of oral care products						
	Use more than one	-	-	1.97 (1.27–3.07)	0.003	1.50 (1.00–2.25)	0.053
	Disabled	-		1.00		1.00	
	Experience of oral symptoms						
	Yes	-	-	1.49 (0.97–2.28)	0.066	1.94 (1.21–3.11)	0.006
	No	-		1.00		1.00	

* The data were analyzed using a complex samples logistic regression, adjusting for sex, grade, academic performance, economic status, father’s education, mother’s education, and mother’s nationality.

## Data Availability

The raw data from the Korea Youth Risk Behavior Survey (KYRBS) can be downloaded from the Korea Disease Control and Prevention Agency. https://www.kdca.go.kr/yhs/ (accessed on 29 January 2024).

## Supplementary Materials

The following supporting information can be downloaded at: https://www.mdpi.com/article/10.3390/healthcare12212141/s1, Variables.
