# Supplementary material for: Factors Associated with Dental Care Utilization for Oral Disease Prevention Among Adolescents in Multicultural Families in Republic of Korea
Source: Healthcare (Basel). 2024 Oct 28;12(21):2141. doi: 10.3390/healthcare12212141 (PMC11545048; doi:10.3390/healthcare12212141)
Supplement: Supplementary file 1 [file healthcare-12-02141-s001.zip › healthcare-3254195-supplementary.pdf]

Supplementary Materials. Variables

Variables		Questions from KYRBS	Categories
**Dependent variables**			
Experience of an asymptomatic dental visit		"Have you experienced any oral symptoms in the last 12 months?" and "Have you had any dental care (including oral examinations at a dental or health clinic) in the last 12 months?"	1)Yes 2) No
Sealant experience		"Have you received sealants in the last 12 months?"	1)Yes 2) No
Scaling experience		"Have you received scaling in the last 12 months?"	1)Yes 2) No
Demographics	Sex		1)Male 2) Female
	Grade	"What grade are you in?"	1) 1st year of middle school<br>2) 2nd year of middle school<br>3) 3rd year of middle school<br>4) 1st year of high school<br>5) 2nd year of high school<br>6) 3rd year of high school
Socioeconomic factors	Academic performance	"How has your academic performance been over the past 12 months?"	1) High 2) Middle 3) Low
	Economic status	"How would you describe your family's economic status?"	1) High 2) Middle 3) Low
	Father's education	"What is your father's highest level of education?"	1) Less than a high school graduate<br>2) College degree or higher
	Mother's education	"What is your mother's highest level of education?"	1) Less than a high school graduate<br>2) College degree or higher
	Mother's nationality	"In which country was your mother born?"	1) China 2) Vietnam

			3) Philippines<br>4) Other
Health perception factors	Subjective health status	"How would you rate your overall health?"	1) Healthy 2) Normal 3) Unhealthy
	Subjective oral health satus	"How would you rate your oral health, including your teeth and gums?"	1) Healthy 2) Normal 3) Unhealthy
Health behavior factors	Fruit consumption	"In the past 7 days, how often did you consume fruit (excluding fruit juice)?"	1) At least once per day<br>2) Less than once per day
	Vegetables consumption	"In the past 7 days, how often did you consume vegetables (excluding kimchi)?"	1) At least once per day<br>2) Less than once per day
	Sugary drinks consumption	"In the past 7 days, how often did you consume sweetened beverages?"	1) Less than 3 times per week<br>2) At least 3 times per week
	Alchol drinking experience	"Have you ever consumed more than one alcoholic beverage?"	1) No 2) Yes
	Smoking experience	"Have you ever smoked even a few puffs of a regular cigarette?"	1) No 2) Yes
Oral health behavioral factors	Daily brushing frequency	"How many times did you brush your teeth yesterday?"	1) Less than 1 time<br>2) More than 2 times
	Brushing before bedtime	"Did you brush your teeth before going to bed yesterday?"	1) Yes 2) No
	Brushing after lunch	"In the last 7 days, how often did you brush your teeth after lunch at school?"	1) Yes 2) No
	Use of oral care products	"In addition to toothpaste and toothbrush, please list any other items you currently use for oral health."	1) Use more than one<br>2) Disabled
	Experience of oral symptoms	"In the past 12 months, have you experienced any of the following symptoms?"	1) Yes 2) No
